# The impact of wound pH on the antibacterial properties of Medical Grade Honey when applied to bacterial isolates present in common foot and ankle wounds. An in vitro study

**DOI:** 10.1186/s13047-023-00653-9

**Published:** 2023-10-02

**Authors:** Carla McArdle, Shirley Coyle, Derek Santos

**Affiliations:** 1 Health Service Executive, St Clare’s Integrated Care Centre, 502 Griffith Avenue, Glasnevin, D11 AT81 Dublin 11 Ireland; 2 Queen Margaret University, Queen Margaret Drive, Musselburgh, EH21 6UU Edinburgh UK

**Keywords:** DFUs, Post-surgical wounds, Medical Grade Honey, Wound pH, Infection, Bacteria, Antibacterial

## Abstract

**Background:**

Diabetic foot ulcers (DFUs) and post-surgical wound infections are amongst the most troublesome complications of diabetes and following foot and ankle surgery (FAS) respectively. Both have significant psychosocial and financial burden for both patients and the healthcare system. FAS has been reported to have higher than average post-surgical infections when compared to other orthopaedic subspecialties. Evidence also indicates that patients with diabetes and other co morbidities undergoing FAS are at a much greater risk of developing surgical site infections (SSIs). With the growing challenges of antibiotic resistance and the increasingly high numbers of resilient bacteria to said antibiotics, the need for alternative antimicrobial therapies has become critical.

**Aim:**

The aim of this study was to investigate the use of medical grade honey (MGH) when altered to environments typically present in foot and ankle wounds including DFUs and post-surgical wounds (pH6-8).

**Methods:**

MGH (Activon) was altered to pH 6, 7 and 8 and experimental inoculums of *Pseudomonas aeruginosa* (NCTC10782), *Escherichia coli*, (NCTC10418), *Staphylococcus aureus* (NCTC10655) and *Staphylococcus epidermidis* (NCTC 5955) were transferred into each pH adjusted MGH and TSB solution and the positive and negative controls.

**Results:**

MGH adjusted to various pH values had the ability to reduce bacteria cell survival in all pH variations for all bacteria tested, with the most bacterial reduction/elimination noted for *Staphylococcus epidermidis*. No correlations were noted among the pH environments investigated and the colony counts, for which there were small amounts of bacteria survived.

**Conclusion:**

This research would indicate that the antibacterial properties of honey remains the same regardless of the pH environment. MGH could therefore potentially be considered for use on non-infected foot and ankle wounds to reduce the bacterial bioburden, the risk of infections and ultimately to improve healing outcomes.

**Supplementary Information:**

The online version contains supplementary material available at 10.1186/s13047-023-00653-9.

## Background

DFUs and postoperative superficial and deep wounds can cause adverse outcomes for patients leading to hospital admissions, delayed healing, poor outcomes, pain, amputations, and death [1, 2]. One in four patients with diabetes will develop a DFU in their lifetime with 50% of those with a DFU developing an infection [3]. A DFU has a major impact on physical functioning, morbidity and exact a high human and financial cost, with cost exceeding that of lung, prostate and breast cancer collectively [4].

Similarly SSIs are among the most common and most expensive health care–associated infections and result in a significant psychosocial and financial burden for both patients and the healthcare system [5]. They have been reported to represent 31% of all hospital acquired illness and are the most common nosocomial infection [6]. Foot and ankle surgery (FAS) has been reported to have higher than average post-surgical infections when compared to other orthopaedic subspecialties [7, 8], with SSI being one of the most troublesome complications after FAS [9]. Post FAS SSI can lead to serious consequences including bone union related issues and joint dysfunction [10]. Additionally, the presence of diabetes complications (defined as the presence of PVD and/or neuropathy) and the presence of neuropathy in non-diabetes patients has been shown to further increase the risk of SSIs following FAS compared with the risks for patients with or without diabetes and/or neuropathy [10]. Wukich and colleagues [10] reported a 7.25-fold increased risk of SSI in patients with complicated diabetes compared with patients without diabetes. Pin site infections are also a well-documented complication of external fixation for correction of Charcot deformity in-patient with diabetes, with infection rates of up to 40% [11].

*Staphylococcus aureus* (S. aureus), Gram-positive cocci, is a major human pathogen and a predominant cause of SSIs [12, 13]. In a relatively recent research study on the bacteriological profile of SSIs, *S. aureus* had a prevalence of over 50% and Gram-negative isolates comprised 49.6% of all aerobic bacterial isolates, *with Escherichia coli (E. coli)* being the most common Gram-negative bacteria, followed by *Pseudomonas aeruginosa* (*P. aeruginosa*). Similar bacteriological profiles have been identified for chronic wounds including DFUs with S. aureus being the predominant bacterial species and the most frequently identified pathogen being P. aeruginosa [14]. Co-infection with *P. aeruginosa* and *S. aureus* is believed to express virulence factors and surface proteins affecting wound healing [15].

Infectious diseases globally are still the second biggest cause of morbidity and mortality due in part to the increase in drug resistance among large numbers of common infecting organisms [16]. For all antibiotic classes, including the major last resort drugs, resistance is increasing worldwide, which poses a serious threat to public health [17]. Increasingly more alarming is the fact that few new antibiotics have been developed in recent decades [18]. Controversy exists around the efficacy of routine use of perioperative antibiotics to prevent infection with evidence indicating that this does not affect wound complications or infection rate [1, 19]. There is therefore a need to investigate additional alternative strategies for wound management to improve patient outcomes through the prevention of infection, decreasing the need for antibiotic therapy [20].

Investigating various aspects of the wound environment and the alterations that occur during the various stages of the healing process may be the way forward for detecting new strategies to reduce the risk of infection. Wound pH has a significant role to play in both directly and indirectly affecting the cellular processes in the wound and has been shown to be one of the critical factors involved in the wound healing process of both chronic and acute wounds [21]. Wound pH is believed to affect matrix metalloproteinase activity, keratinocyte proliferation, fibroblast activity, microbial proliferation, biofilm formation and immunological responses [18]. Strohal and colleagues in their pilot study on 30 wounds identified that wounds, which presented with a highly alkaline pH (9) at the start of the study progressed to the mean pH decreasing significantly over time with the ulcers having an almost neutral pH as the wound progressed towards healing [22]. Researchers reported that decreased wound size correlated significantly with a reduction in the pH of the wound [22]. In the same study [22] successful control of infection and a reduction in bioburden correlated with a statistical signification pH change from an alkaline towards an acidic wound environment. Agrawal and colleagues in their study on 100 infected wounds similarly noted the role of pH in wound healing with an acidic pH inhibiting the growth of bacteria [23]. Shukla and colleagues [24] in a much earlier study concurred with the aforementioned. They observed that improvements in wound status was associated with reductions in wound pH with decreases during the study period being associated with wounds progressing from ‘unhealthy’ towards a ‘granulating’ or ‘healthy’ status.

Research to date could thus indicate that lowering wound pH may provide an opportunity to create an environment, which allows the wound to progress towards healing. Medical grade honey (MGH) known to have a pH of four may potentially halt the growth of most common bacteria [22, 23, 25]. Honey is believed to present with high levels of antimicrobial compounds including methylglyoxal and bee defensin-1, and glycoside derivatives, all known to effectively inhibit viable bacteria of resistance strains [16, 25, 26]. Due to the antimicrobial effects of honeys from the simultaneous action of pH coupled with the many active compounds present, bacteria are deemed unlikely to develop resistance to this substance [27]. Additionally, its ability to accelerate wound healing and its low-cost production make it an attractive option in wound care [28].

Topical agents and wound dressings form an important part of all wound management plans regardless of wound aetiology, and their therapeutic availability has increased tenfold in the last decade [29]. While the aforementioned research indicates that the wound pH changes during the healing process and during times of infection, it does however remain unclear if wound pH affects the efficacy of such topical agents like MGH (Activon).

### Aim

The aim of this research is to investigate the antibacterial effects of MGH when altered to pH environments known to exist in wounds prevalent to the foot and ankle including DFUs and post-surgical wounds and exposed to typical bacteria commonly found in said wounds.

### Methods

The in vitro research used a broth culture assay of Tryptone Soya Broth (TSB, Oxoid, Basingstoke, UK), a well characterised and standardised medium known to support the growth of the below organisms) and honey (sterile medical grade Manuka honey) (Advancis Medical, UK) adjusted to pH values known to exist in wounds (pH 6, pH 7 and pH 8). This broth culture was used to investigate the effects of pH changes and honey on the growth of Gram-negative and Gram-positive bacterial species/strains isolated from post-surgical wounds.

### Microorganisms used for the research

Common foot and ankle wound pathogens were sourced from the National Collection of Type Cultures (NCTC) and isolated from wounds. These included; *P. aeruginosa* (NCTC10782), *E. coli*, (NCTC10418), *S. aureus* (NCTC10655) and *Staphylococcus epidermidis* (*S. epidermidis*) (NCTC5955). Stock cultures were created by inoculating 10 ml of Tryptone Soya Broth (TSB) (product code: CM0129) with 100 µL of said bacterial isolates which were incubated overnight.

### Dispensing bacteria into each solution

Experimental inoculums were obtained by transferring 100 µL of approx. 10^12^ CFUs/ml of *P. aeruginosa, E. coli, S. aureus, and* approx. 10^9^ CFUs/ml of *S. epidermidis* individually into each pH adjusted honey and TSB solution and the positive and negative controls. Single celled communities of bacteria were investigated to explore if the presence of said organisms had an impact on the antibacterial properties of honey.

### Liquid and solid media preparation

TSB and Tryptone Soya Agar (TSA) (product code: CM0003), and Phosphate Buffered Saline (PBS) solution (product code: BR0052) (Oxoid, Basingstoke, UK) was prepared by suspending the dry ingredients in double distilled water. Following this, the broths and agars were brought to boiling point and dispensed into tubes or bottles, then heated at 121 °C for 15 min in an autoclave to ensure sterilisation. TSA is prepared by a similar method, but aseptically dispensed into sterile disposable plastic Petri dishes while still molten and allowed to set overnight to form sterile agar plates.

### pH adjusted TSB and Medical Grade Honey (MGH)

TSB was prepared to a clinically relevant range of final pH values of pH 6, pH 7 and pH 8 using hydrochloric acid and sodium hydroxide (Fisher, UK). Activon has been chosen for this study as it contains 100% sterile MGH and is a currently used topically on wounds within the National Health Service (NHS) to aid in the healing process. A method previously described by Schneider et al. [30] with final honey concentrations of 75% (w/v) honey was used for this research. Briefly, 7.5 g of MGH was added to TSB to a final solution of 10mls. Due to honey having an acidic pH (4), the pH of the TSB and honey solution was measured with an electronic pH meter (Fisher Scientific Accumet AE150) and either hydrochloric acid or sodium hydroxide was added accordingly in order to gain the optimum pH values (pH 6, 7 and 8). Following this, 100 µL of the experimental inoculums as explained above of each single bacteria was aseptically dispensed into the TSB and honey solutions and incubated in an orbital incubator at 100 RPM for 24 h at 37 °C.

### Controls used in the experiment

Controlled experiments ran concurrently with the above pH adjusted TSB and honey solutions. The controls included pH altered TSB (with no honey) with final pH values of pH 6, pH 7 and pH 8 as well as the natural occurring pH value of honey (pH 4). As stated above, the pH was altered using hydrochloric acid and sodium hydroxide (Fisher, UK). A further positive control using honey without pH adjustment and a negative control of TSB with no bacteria or honey was also used. One hundred microliters of 10^8^ CFUs/ml of each organism were aseptically dispensed into all controls and incubated for 24 h at 37 °C.

### Serial dilutions and plate counts

Serial dilutions and plate counts are the most frequently used method for estimation of bacterial numbers [31]. In this procedure, bacterial cultures or suspensions are serially diluted in 9 ml isotonic diluent. Triplicate samples were used from each member of the dilution series (containing single cells), spreading cells over the surface of all agar plates (e.g. Tryptone Soya Agar, TSA, Oxoid). The plate were then incubated for 24 h, and individual cells or clumps (defined as colony forming units, cfu) multiplied to form optically visible and countable colonies on the agar surface. Surviving viable cell numbers were estimated by counting agar plates with CFUs between 30 and 300. The reason for being, if there are too few colonies (< 30), the count may not be accurate and too many colonies (> 300) it is difficult as well as time-consuming to distinguish the individual colonies on a plate [32]. Multiplication of the number of colonies by the dilution factor provides an estimate of the bacterial number in the original culture/suspension, usually reported as log_10_ CFU/ml [31].

### Data analysis

All tests were carried out in triplicate, with the negative control and altered honey and pH experiments being repeated on three separate occasions. The data was recorded as mean ± standard error of the mean and was analysed in Microsoft excel 2016. The data was compared to the relevant controls using a two-tailed independent student t-test as used in previous research [25]. A *p* value of ≤ 0.05 was considered statistically significant.

## Results

**Fig. 1:**
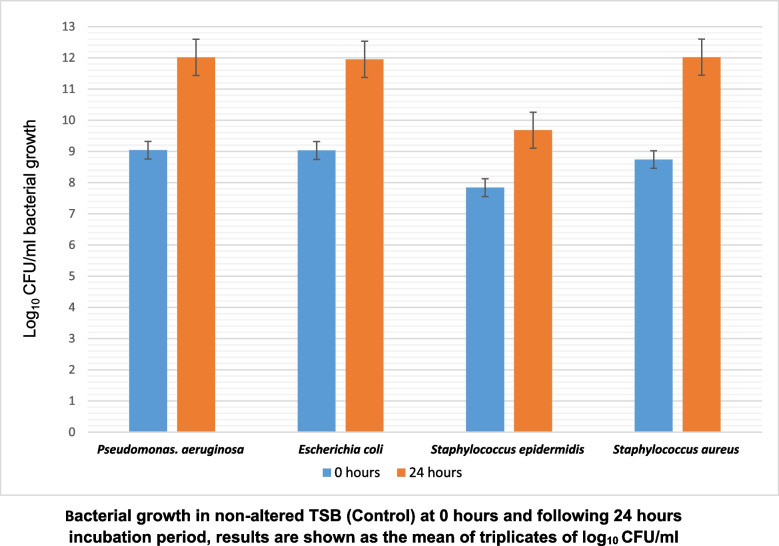
Bacterial growth in non-altered TSB (Control) at 0 hours and following 24 hours incubation period. All results are shown as the mean of triplicates log10 CFU/ml

Four clinically relevant isolates commonly found in foot and ankle wounds were investigated including; *P. aeruginosa, E. coli, S. aureus* and *S. epidermidis*. Initially, all bacterial isolates were inoculated in non-altered TSB and incubated for 24 h as a negative control. The results of all colony counts that were performed at 0 h and 24 h are shown in Fig. 1. All bacterial colony counts increased over 24 h with average growth (log) counts of 10^3^ CFU/ml. Bacterial growth at 24 h reached 10^9^ CFU/ml for *S. epidermidis* and 10^12^ CFU/ml for *P. aeruginosa, E. coli* and *S. aureus*.

**Fig. 2:**
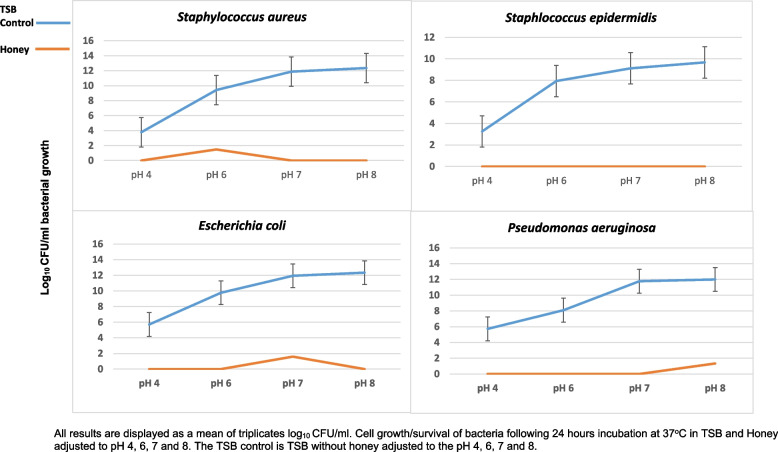
Bacterial growth/survival following 24 h incubation at pH altered Honey and TSB and in the TSB control

One hundred microliters of the wound bacterial isolates after 24 h of incubation were added to solutions of honey with altered pH values, and the positive control of TSB with the same altered pH values. The results of the experiment performed are shown in Fig. 2 and Table 1. Results show that 75% (w/v) MGH present in all pH environments investigated can reduce bacteria to undetectable amounts with *S. epidermidis*, and less than 10^2^ CFU/ml with *P. aeruginosa*, *E. coli* and *S. aureus.*

**Table 1:**
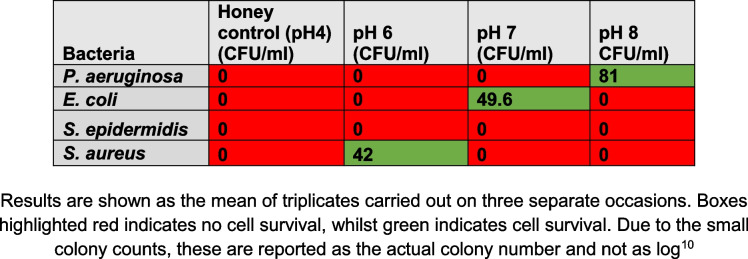
Cell survival (CFU/ml) after 24-h incubation period with Medical Grade Honey (Activon) in various pH environments

More specifically, for *S. epidermidis,* honey reduced the bacterial cell survival by an excess of 10^9^ CFU/ml for all investigations regardless of the pH environment (*p* ≤ 0.5). For *P. aeruginosa, E. coli* and *S. aureus* cell survival reduced by at least 10^10^ CFU/ml and as much as 10^12^ CFU/ml in all investigations regardless of the pH environment (*p* ≤ 0.5). *S. aureus, E. coli and P. aeruginosa* had some bacteria survival at pH 6, 7 and 8 respectively, however these were of extremely small quantities with all final colony counts averaging less than 10^2^ CFU/ml after three runs of each experiment. There were no correlations or statistical significance noted among the pH environments investigated and the colony counts that had small amounts of bacterial survival.

Results are shown as the mean of triplicates carried out on three separate occasions. Boxes highlighted red indicates no cell survival, whilst green indicates cell survival. Due to the small colony counts, these are reported as the actual colony number and not as log.^10^

When comparing the cell growth/survival of bacteria in honey and pH environments of pH 6, 7 and 8 to that of the growth media TSB at the same altered pH environments, distinct differences were identified (Fig. 1) which were all statistically significant (*p* ≤ 0.05). Cell survival/growth at all pH environments and TSB exceeded colony counts of 10^8^ CFU/ml. Alkaline pH values (pH 7 and 8) had a greater bacterial growth, increasing to as much as 10^12^ CFU/ml for *P. aeruginosa, E. coli and S. aureus* and close to 10^10^ CFU/ml for *S. epidermidis.* For all bacteria investigated, pH 8 demonstrated to have the highest bacteria survival when compared to all other pH environments. Contrary to this, the acidic environment of pH 4 for all bacteria was identified as having the least bacterial survival with colony counts for Gram-positive bacteria (*S. aureus* and *S. epidermidis)* being 10^4^ CFU/ml*.* Gram-negative bacteria *(P. aeruginosa* and *E. coli)* demonstrated slightly more resilience at pH 4 with survival colony counts reaching as high as 10^6^ CFU/ml. Figure 2 illustrates these differences with log numbers increasing as pH moves from acidic to alkaline.

Collectively, all results demonstrated that honey regardless of pH environment eliminated bacteria to undetectable levels in the majority of experiments. When bacterial survival was noted, (Table 1 and Fig. 1) extremely small quantities (< 10^2^ CFU/ml) were calculated on the neat plates i.e. not requiring a serial dilution due to the low quality that remained. However, when bacteria was added to pH altered TSB (growth media), bacterial growth increased as the pH became more alkaline for all Gram-negative and Gram-positive bacterial isolates. The results show that bacterial growth favours alkaline pH environments and therefore growth is hindered in acidic pH environments.

## Discussion

This in vitro study investigated the impact of pH on the ability of MGH to eliminate bacteria known to present in wounds of the foot and ankle. The results indicated that MGH plays a key role in the metabolic disruption and elimination of Gram-negative and Gram-positive bacteria. Throughout the research, the role of honey in woundcare has been extensively documented, with honey being applied to wounds for centuries because of its broad-spectrum antimicrobial and wound healing activities [33, 34]. Current research has found the antimicrobial and healing ability of honey to be attributed in part to the acidic pH. Believing that an acidic pH makes it harder for bacteria to persist, while also speeding up the healing process through increasing the amount of oxygen offloaded from haemoglobin in the capillaries and the suppression of protease activity [35–38]. While the acidification of the wound environment is an important mechanism by which honey induces healing [38], it would appear from the results of this investigation that the other properties of honey provide equally vital antimicrobial activities which go beyond pH. Nair and colleagues [33] describes MGH as consisting of more than 200 different constituents, of which water and carbohydrates such as glucose, fructose and sucrose encompass the relative majority. Moreover, other molecules present in MGH, which also have a direct antimicrobial effect including; phenolic compounds, hydrogen peroxide, flavonoids, methylglyoxal and a bee-originating enzyme called glucose oxidase [34, 39]. In addition phenolic compounds, organic acids, vitamins, and flavonoids provide honey with antioxidants and anti-inflammatory properties which boosts the antibacterial effects [40].

To date, this is the first in vitro research to focus on altering the pH of honey to assess the impact on bacterial survival of clinically relevant organisms specific to foot and ankle wounds. A previous study by McArdle et al. [41] investigated the resistance of bacteria isolated from DFU wounds to antibiotics frequently used in the management of wound infection, at similar pH ranges to this study (pH 6.5–8.5). Authors identified that alterations to pH subsequently modified the resistance of bacteria when exposed to common clinically relevant antibiotics. While this study [41] did not investigate the use of honey, as a comparison to the current i*n vitro* study findings it would indicate that honey can withstand subtle changes in pH where antibiotics cannot.

Antibiotic resistance has been described as one of the greatest challenges the world faces [17]. Indiscriminate use of antibiotics and growing numbers of resistant strains will considerably impact patient’s morbidity and mortality in particular when we relate this to SSIs and diabetic foot infections [42]. It is estimated that in 2050, resistance will account for 10 million extra deaths annually worldwide, with additional catastrophic economic costs [43, 44]. The increasing tolerance of biofilm to both systemic antibiotics and topical antiseptics mandate consideration of the addition of anti-biofilm strategies to any accepted protocol of care for both DFUs and DFIs [45]. Despite all the beneficial effects of MGH which this current investigation can agree with, its use is often reserved for further down the line of therapy due to clinicians tending to stick to conventional treatments such as iodine and antibiotics [33]. A retrospective analysis on 641 patients undergoing elective FAS investigated the use of antibiotics postoperatively. Researchers identified that there was no significant difference between the numbers of infections in the group with postoperative antibiotics and the group without, with authors identifying that the routine use of postoperative antibiotics does not affect wound complications or infection rate [1]. Therefore, using MGH instead of antibiotics as an earlier postoperative wound treatment intervention may therefore enhance healing, prevent infection and decrease the possibility of antibiotic resistance.

Due to the multi-drug resistance nature of *P. aeruginosa* infections, these are currently treated using combinations of beta-lactams, fluoroquinolones, and aminoglycosides [46]. The diverse virulence factors also participate in the development and spread of the infection associated with higher health costs and mortality rates, as well as longer hospital stays and treatment courses [46]. Mohammadzamani and colleagues [46] investigated the inhibitory effects of other alternatives to antibiotics which included, cinnamon (Cinnamaldehyde), thyme (Carvacrol), and honey on the expression of genes in 35 multi-resistant *P. aeruginosa* isolates recovered from burn wound infections using a control antibiotic (Imipenem) for comparison. The findings of the research [46] can somewhat be compared to authors’ in vitro study as they too found honey to be useful as an early alternative to antibiotic therapy. The study [46] however went a step further with their findings and found that the minimum inhibitory concentration of a combination of Cinnamaldehyde, Carvacrol, and honey was 100 times lower compared to that of the antibiotic tested (Imipenem). Thus, indicating that the antimicrobial properties of a combination of therapies can provide a much greater ability to reduce the risk of infection and offer a further appropriate alternative to treat bacterial infections.

A further study showing clinical comparisons with the authors in vitro study is that of Lazarides and colleagues [47]. Researchers in this prospective case series, which included 19 patients with diabetes with a Charcot foot undergoing external fixation, applied honey (Medihoney® patches) to pin sites at the end of the procedure, replacing weekly for a total of 8 weeks. Patients were monitored for infections from the time of the surgery until external fixator removal. In addition to the prospective group, Lazarides and colleagues included a control group of 16 consecutive patients with diabetes who received standard pin care post surgically i.e. did not include honey, and evaluated medical records for pin site complications and infections. While the study numbers are low and the retrospective nature of the control group is a limitation to the study, researchers did however observe a statistically significant difference in pin site infections. The control group had pin site infections in 9 patients compared to 2 patients in the honey group indicating that the use of honey reduces the risk of infection. The results of the authors’ in vitro study would further explain why infection presented less in the honey group, with this being attributed to the fact that honey has the ability to reduce bacterial cells to undetectable levels regardless of the pH it presents in.

Occasionally in medicine, wound infection is diagnosed by quantitative bacteriology whereby there is bacterial cultures present of > 10^5^ CFU/g tissue [48], alongside the classic signs of infection determined by clinical judgement. Through applying this quantitative bacteriology to this in vitro study, it would suggest clinically that MGH could reduce bacteria in all cases to levels that would not indicate wound infection. A further suggestion would be that this is the case regardless of the pH environment that the MGH and bacteria present in.

To date, no clear consensus has been reached regarding how rapidly bacteria exposed to honey can evolve reduced susceptibility to it [49]. Researchers believe that honey displays low propensity for resistance, attributed to its complexity acting in a multifactorial way to target cells via several antibacterial compounds [27, 50, 51]. Cooper and colleagues [51] explains that permanent honey resistant mutants will be rare if high concentrations of Manuka honey are maintained in practice. Clinically for wounds, this means regular dressing changes to help keep levels high, particularly in highly exuding wounds. Lu et al. [52] in a later study highlights that honey resistant bacteria research to date has only been explored in planktonic cells and not within biofilm cells. Authors explored the potential for honey resistance in strains of *P. aeruginosa,* of which they found similar findings to previous research. More specifically, authors identified that honey eradicated *P. aeruginosa* biofilms but if biofilms are treated with lower concentrations *P. aeruginosa* showed slightly increased tolerance to honey [52]. Lu et al. [52] concurs with the findings of this in vitro study for *pseudomonas* as authors too found that honey had the ability to eliminate bacterial strains to an undetectable level. It is important to note however, that the current research does not consider the biofilm formation of bacteria investigated. These findings emphasise the use of MGH as a potential first line woundcare treatment modality in order to reduce pathogenic pseudomonas species in DFUs and postoperative wounds, thus reducing the risk of infection developing.

This in vitro study can in part address a noteworthy question raised by Cooper and colleagues [53] which relates to whether the pH of honey applied to wounds changes, and if said changes has an impact on the antibacterial properties of honey. Cooper et al., explained that the role of honey’s pH might be limited when added to body fluids, which are buffered, as the pH will not be as low and therefore the acidity of honey may not be as effective at inhibiting growth of many species. Body pH is stabilised by a protein buffering system present in body fluids, which has the ability to bind or release H + in solution, thus keeping the pH of the solution relatively constant despite the addition of considerable quantities of acid or base. This buffering system is plentiful in blood and tissue cells. However, this it believed not to be the case with wound fluid of all wounds of mixed aetiologies, which includes that of surgical wounds [21, 24]. While applying MGH to a wound bed with fluids may alter the pH, the findings of the authors’ in vitro study would suggest that the pH of the wound fluid and subsequent pH of honey has no impact on its ability to reduce bacterial load of the Gram-positive and Gram-negative bacteria investigated. Although this finding needs to be confirmed clinically.

In recent years, greater interest is evident for evaluating the effects of honey on antibiotic resistant organisms, as well as the use of honey as a wound management alternative to antibiotic therapy and other conventional wound dressings [38]. The findings of this investigation can concur with in vitro work by Wadi [54] as they too found honey to be effective at reducing bacterial bioburden for clinical isolates similar to those found in foot and ankle wounds including *S. aureus, E. coli* and *P. aeruginosa*. Authors [54] however tested various different honey samples and did not alter pH. They did however compare their findings to common clinically used antibiotics and identified that honey was more effective at controlling bacterial growth [38].

The findings of this study alongside the findings of similar aforementioned research carried out within the area [33, 46, 52, 54], would suggest that MGH should be considered as the treatment of choice for foot and ankle wounds of mixed aetiology regardless of the presenting pH environment. MGH has been shown to reduce bacterial load of commonly found bacteria isolates to undetectable levels, a key to preventing infection and thus aiding with the healing process.

## Limitations and future research

While this study has significant clinical relevance, it does however present with a number of limitations. The first being the in vitro nature of the study which is a limitation, future research should investigate the clinical use of MGH when applied to various wound pH environments and if the repeated application of MGH can maintain a low pH on the wound bed.

Within this study honey was investigated at a single concentration (75% (w/v) in TSB), it would be interesting and necessary for future research to focus on other concentrations i.e. 25% and 50% in order to establish the minimum inhibitory concentration for honey and if the various pH environments explored in this study have an impact on cell survival. Cell survival of bacteria was investigated as single celled colonies in the cells planktonic form and not in biofilms. Future research could investigate more resistant bacterial strains for example, MRSA and *Streptococcus Pyrogenes*. While also considering the antibacterial properties of honey when in biofilm cells both in single celled colonies and in polymicrobial colonies to more truly reflect that of the wound bed.

## Conclusion

Collectively the findings of this research indicate that MGH regardless of pH environments can reduce bacteria to undetectable amounts with *S. epidermidis*, and to extremely small amounts (less than 10^2^ CFU/ml) with *P. aeruginosa, E. coli* and *S. aureus*. Clinically, these results would support the continued use of MGH in medicine to prevent infection in wounds. More specially, the application of MGH to DFUs and following foot and ankle surgical procedures should be considered as a prophylactic measure for wound infection and to aid in the wound healing process. MGH can also be considered as a replacement to prophylactic antibiotics and other conventional wound management modalities.

Infected wounds can be devastating for the patients, and the increase in bacterial resistance to common antibiotics is a global challenge hitting crisis point, therefore new strategies for reducing both is sorely required. The concentration used in this in vitro study was shown to be enough to reduce bacterial bioburden either to undetectable levels or to a quantity unlikely to cause infection. The current findings concur with previous research that indicates that regular application of honey to the wound bed can provide continued antibacterial effects. Future research should investigate other concentrations to establish if the pH has a greater impact at lower quantities and if various honey concentrations have an impact on biofilm cells, polymicrobial in nature. While also including more resistance bacterial strains including MRSA and *Streptococcus Pyrogenes.*

### Supplementary Information

**Additional file 1.**

## Data Availability

All data generated or analysed during this study are included in this published article and its supplementary information files.

## Abbreviations

DFU: Diabetic foot ulcer
E. coli: *Escherichia coli*
FAS: Foot and Ankle Surgery
MGH: Medical Grade Honey
P. aeruginosa: *Pseudomonas aeruginosa*
S. aureus: *Staphylococcus aureus*
S. epidermidis: *Staphylococcus epidermidis*
SSI: Surgical Site Infection
TSA: Tryptone Soya Agar
TSB: Tryptone Soya Broth
